# Supplementary material for: PON1 Status in Relation to Gulf War Illness: Evidence of Gene–Exposure Interactions from a Multisite Case–Control Study of 1990–1991 Gulf War Veterans
Source: Int J Environ Res Public Health. 2024 Jul 24;21(8):964. doi: 10.3390/ijerph21080964 (PMC11353671; doi:10.3390/ijerph21080964)
Supplement: Supplementary file 1 [file ijerph-21-00964-s001.zip › ijerph-3058722-supplementary.pdf]

International Journal of
Environmental Research and Public Health

MDPI

Table S1. Mean (and median) PON1 enzyme activity in three substrates by PON1 status.

	All Veterans (n=398)	QQ Status (n=169)	QR Status (n=172)	RR Status (n=57)
**Mean (median) Paraoxonase Activity**: All Veterans	723.2 (646.7)	304.2 (295.7)	911.0 (897.7)	1398.6 (1436.8)
GWI Cases Only	720.8 (642.6)	297.2 (293.5)	902.5 (868.5)	1433.6 (1487.6)
Controls Only	710.9 (667.9)	324.2 (322.5)	934.2 (956.0)	1280.3 (1321.3)
(Cases vs. controls [a]*)*	$p = 0.797$	$p = 0.201$	$p = 0.505$	$p = 0.242$
**Mean (median) Arylesterase Activity**: All Veterans	134.8 (132.3)	142.6 (139.7)	132.7 (127.0)	119.2 (128.8)
GWI Cases Only	133.2 (129.7)	139.8 (138.8)	132.7 (124.7)	121.7 (128.8)
Controls Only	135.8 (132.5)	151.4 (146.9)	132.8 (131.1)	110.6 (127.8)
(Cases vs. controls [b]*)*	$p = 0.569$	$p = 0.169$	$p = 0.985$	$p = 0.442$
**Mean (median) Diazoxonase Activity:** All Veterans	9658.8 (9315.2)	11,542.1 (11,402.5)	9284.8 (8946.9)	5203.7 (5098.9)
GWI Cases Only	9631.8 (9407.5)	11,584.0 (11,442.1)	9319.9 (9071.1)	5369.2 (5256.2)
Controls Only	9485.0 (9263.7)	11,423.2 (11,309.1)	9188.5 (8656.3)	4643.8 (4437.9)
(Cases vs. controls [b]*)*	$p = 0.783$	$p = 0.795$	$p = 0.846$	$p = 0.279$

Enzyme activity in designated substrate (in U/mL); [a] Wilcoxon-Mann-Whitney test; [b] *t*-test.

Table S2. PON1 status and enzyme activity over time among San Francisco Gulf War veteran subjects (n=24) assayed twice.

	SF DOD study	SF VA study
Years when PON1 was assayed	2002-2006	2017-2018
PON1 Status Distribution:	n (%)	n (%)
QQ	13 (54%)	13 (54%)
QR	10 (42%)	10 (42%)
RR	1 (4%)	1 (4%)
Mean (Median) PON1 enzyme activity [a] (units/mL)		
Paraoxonase	704.4 (520.2)	528.3 (432.4) [b]
Diazoxonase	11,480.5 (10,292.8)	8441.6 (9140.8) [b]

Abbreviations: SF = San Francisco; DOD = U.S. Department of Defense, VA = U.S. Department of Veterans Affairs; [a] in designated substrate; [b] significantly different from earlier timepoint, paired t-test and Wilcoxon Signed-Rank test, $p<0.001$.

Table S3. Spearman's correlation (ϱ) between deployment related exposures (ever exposed).

	Smoke	Alarm	SCUD	Ground	POW	Vehicle	Skin Pest	Uniform	Flea	Fogging	PB
**Smoke**	--										
**Alarm**	0.29 [c]	--									
**SCUD**	0.15 [c]	0.32 [c]	--								
**Ground**	0.21 [c]	0.23 [c]	0.01	--							
**POW**	0.34 [c]	0.31 [c]	0.17 [c]	0.49 [c]	--						
**Vehicle**	0.33 [c]	0.32 [c]	0.07	0.52 [c]	0.52 [c]	--					
**Skin Pest**	0.19 [c]	0.36 [c]	0.17 [c]	0.21 [c]	0.29 [c]	0.35 [c]	--				
**Uniform**	0.14 [b]	0.29 [c]	0.17 [c]	0.20 [c]	0.23 [c]	0.24 [c]	0.44 [c]	--			
**Flea**	0.06	0.08	0.10 [a]	0.17 [c]	0.13 [c]	0.07	0.23 [c]	0.22 [c]	--		
**Fogging**	0.16 [c]	0.19 [c]	0.14 [b]	0.08	0.15 [b]	0.18 [c]	0.35 [c]	0.25 [c]	0.16 [c]	--	
**PB**	0.21 [c]	0.36 [c]	0.14 [b]	0.39 [c]	0.38 [c]	0.37 [c]	0.35 [c]	0.25 [c]	0.11 [a]	0.22 [c]	--

Significant correlations: [a] $p<0.05$, [b] $p<0.01$, [c] $p<0.001$; Abbreviations: Smoke = saw oil fire smoke; Alarm = heard chemical alarms sound; SCUD = being within 1 mile of SCUD missile explosion; Ground = Directly involved in ground combat; POW = contact with prisoners of war; Vehicle = contact with destroyed enemy vehicles; Skin Pest = used spray or cream pesticides directly on skin; Uniform = wore pesticide-treated uniforms; Flea = wore flea collars; Fogging = saw area sprayed/fogged w/ pesticides; PB = used pyridostigmine bromide. pills

Table S4. Association of GWI with potentially cholinergic exposures in pyridostigmine bromide (PB) use subgroups.

	All Veterans (n=398: 295 GWI Cases, 103 Ctrl)			No PB Use vs. Any PB Use: No PB Used (n=89: 48 GWI Cases, 41 Ctrl)			No PB Use vs. Any PB Use: Used PB (n=301: 239 GWI Cases, 62 Ctrl)		
	n (%) Exposed			n (%) Exposed			n (%) Exposed		
**Exposures**	GWI	Ctrl	OR_{adj} [a] (95% C.I.)	GWI	Ctrl	OR_{adj} [a] (95% C.I.)	GWI	Ctrl	OR_{adj} [a] (95% C.I.)
Heard chemical alarms sound [b]	247 (86%)	65 (64%)	2.01 (1.10, 3.66) [d]	27 (56%)	18 (44%)	1.20 (0.44, 3.30)	220 (92%)	47 (77%)	2.42 (1.05, 5.59) [d]
Used cream/spray pesticide on skin [c]	210 (73%)	37 (36%)	3.77 (2.21, 6.42) [e]	20 (42%)	9 (22%)	2.36 (0.77, 7.18)	190 (79%)	28 (45%)	4.00 (2.13, 7.49) [e]
Saw area sprayed/fogged w/ pesticides	106 (38%)	22 (21%)	1.29 (0.70, 2.38)	10 (21%)	5 (12%)	1.27 (0.31, 5.16)	96 (41%)	17 (27%)	1.24 (0.62, 2.47)
Wore uniform treated with pesticides	138 (48%	26 (25%)	1.50 (0.82, 2.76)	9 (19%)	9 (22%)	0.63 (0.17, 2.35)	129 (54%)	17 (27%)	1.85 (0.91, 3.77)
Regular smoker during deployment	66 (23%)	20 (19%)	0.90 (0.48, 1.69)	6 (12%)	8 (20%)	0.61 (0.17, 2.15)	60 (25%)	12 (20%)	0.87 (1.06, 5.79)

Abbreviations: GWI = Gulf War Illness; Ctrl = Controls; PB = Pyridostigmine Bromide; OR = Odds Ratio; C.I. = Confidence Interval; [a] Logistic regression model adjusted for hearing chemical alarms, using pesticide cream/spray on skin, age, rank; [b] In adjusted models, interaction term (hearing chemical alarms X use of PB pills was not significant, p=0.159; [c] In adjusted models, interaction term (using pesticides on skin X use of PB pills) was significant, p=0.031; significant associations: [d] p<0.05, [e] p<0.001.

Table S5. Association of GWI with potentially cholinergic exposures by deployment smoking status.

				Deployment Smoking Status					
	**All Veterans (n=398: 295 GWI Cases, 103 Ctrl)**			**Not Regular Smoker (n=302: 219 GWI cases, 83 Ctrl)**			**Regular Smoker (n=86: 66 GWI cases, 20 Ctrl)**		
	n (%) Exposed			n (%) Exposed			n (%) Exposed		
**Exposures**	GWI	Ctrl	OR_{adj} [a] (95% C.I.)	GWI	Ctrl	OR_{adj} [a] (95% C.I.)	GWI	Ctrl	OR_{adj} [a] (95% C.I.)
Heard chemical alarms sound [b]	247 (86%)	65 (64%)	2.01 (1.10, 3.66) [d]	183 (84%)	53 (65%)	1.89 (0.96, 3.72)	62 (94%)	12 (60%)	6.25 (1.01, 38.76) [d]
Used cream/spray pesticide on skin [c]	210 (73%)	37 (36%)	3.77 (2.21, 6.42) [e]	152 (69%)	33 (40%)	2.64 (1.44, 4.83) [d]	56 (85%)	4 (20%)	23.85 (5.62, 101.21) [e]
Saw area sprayed/fogged w/ pesticides	106 (38%)	22 (21%)	1.29 (0.70, 2.38)	80 (37%)	19 (23%)	1.13 (0.57, 2.25)	26 (40%)	3 (15%)	1.65 (0.33, 8.17)
Wore uniform treated with pesticides	138 (48%)	26 (25%)	1.50 (0.82, 2.76)	104 (47%)	23 (28%)	1.45 (0.74, 2.85)	34 (52%)	3 (15%)	0.83 (0.11, 6.34)
Took PB Pills	239 (83%)	62 (60%)	1.73 (0.94, 3.17)	177 (81%)	50 (60%)	1.90 (0.98, 3.68)	60 (91%)	12 (60%)	0.71 (0.06, 8.89)

Abbreviations: GWI = Gulf War Illness; Ctrl = Controls; PB = Pyridostigmine Bromide; OR = Odds Ratio; C.I. = Confidence Interval; [a] Logistic regression model adjusted for hearing chemical alarms, using pesticide cream/spray on skin, age, rank; [b] In adjusted models, interaction term (hearing chemical alarms X smoking during deployment) was not significant, p=0.125; [c] In adjusted models, interaction term (using pesticides on skin X smoking during deployment) was significant, p=0.004; Significant association: [d] p<0.05, [e] p<0.001.
